# Using ATP measurements to rapidly evaluate the cleanliness of spectacle surfaces

**DOI:** 10.1186/s13104-025-07282-4

**Published:** 2025-05-14

**Authors:** Nazma Sultana Lupin, Birgit Fritz, Siegfried Wahl, Markus Egert

**Affiliations:** 1 Faculty of Health, Medical and Life Sciences, Institute of Precision Medicine, Microbiology and Hygiene Group, Furtwangen University, Villingen-Schwenningen, Germany; 2 ZEISS Vision Science Lab, Institute for Ophthalmic Research, Eberhard-Karls-University, Tuebingen, Germany

**Keywords:** Spectacles, Hygiene, ATP, Rapid method, Cleaning

## Abstract

**Objective:**

Microbial contamination on used spectacles poses a potential health risk to spectacle wearers and people regularly working with worn spectacles, such as opticians. ATP measurement is widely used to evaluate the cleanliness of surfaces based on the detection of adenosine triphosphate, a molecule found in and around living cells. In this study, we investigated the suitability of this method to rapidly and easily evaluate the efficacy of different cleaning methods for spectacles. Additionally, we examined the correlation between ATP content on spectacle surfaces with aerobic and anaerobic bacterial colony counts. We swab-sampled worn spectacles and used an ATP bioluminescence assay to assess the level of cellular contamination.

**Results:**

Six cleaning methods were tested on ten worn spectacles each, and ATP levels were correlated with both aerobic and anaerobic bacterial counts. All investigated cleaning methods showed a significant median reduction of the ATP content on spectacle surfaces, between 75 and 93%. Germ counts and ATP levels showed no significant correlation for aerobic but for anaerobic cultivation. Higher anaerobic germ counts correlated positively with higher ATP levels. ATP measurement is a suitable method to rapidly and easily demonstrate the efficacy of cleaning measures for spectacle surfaces also under non laboratory conditions.

## Introduction

Surfaces that come into regular contact with the human body typically harbour microorganisms, the majority of which are part of the resident skin and mucosa microbiota. Despite their largely beneficial nature for humans, some these microorganisms still possess pathogenic potential. Hence, such surfaces are classified as fomites [1]. Recent studies have investigated the microbial load and potential health risks associated with frequently touched items, including smartphones [2], hospital surfaces [3] and spectacles [4]. Spectacles, ubiquitous optical devices designed to enhance human vision, are positioned prominently on the face, exposed to the environment and in close contact with skin, nose, and mouth. Their regular handling with human hands further emphasizes their potential for microbial contamination. Despite their widespread use, limited research has been conducted on the microbiota of spectacles, leaving a gap in our understanding of potential microbial presence [4]. In clinical settings, spectacles worn by surgeons were recognized as fomites [5]. In our earlier research, we observed that regular cleaning of spectacles has the potential to significantly reduce the microbial load, with a notable decrease of up to 2 log scales [6]. Notably, impregnated lens wipes emerged as the most effective cleaning method, achieving a median germ reduction between 99 and 100%, while dry cleaning showed less efficacy with median germ reduction ranging from 85 to 90% [4].

Adenosine triphosphate (ATP) measurements have a long tradition to monitor the cleanliness of surfaces in different industry sectors and the field of healthcare [7–9]. Although ATP testing has proven to be a highly sensitive method for establishing cleanliness standards in healthcare settings [10], the ATP bioluminescence method is particularly effective for assessing cleaning efficacy and can be integrated into daily clinical practice for routine monitoring. Additionally, it helps identify high-risk surfaces, those prone to rapid re-soiling, and surfaces that are easier to clean [11].

Cultivation of microorganisms is the traditional and widely adopted method for assessing microbial cleanliness and disinfection effectiveness. It is crucial to emphasize that the incubation process for these cultures typically extends over several days [7]. In the last decade, numerous medical institutions such as the US Centers for Disease Control and Prevention (CDCs), and healthcare oversight organizations in China, have increasingly turned to the ATP bioluminescence method to assess surface cleaning and disinfection on-site [12]. The ATP bioluminescence method detects the ATP content in a sample, stemming from living cells, by observing the biological luminescence reaction in a luciferase assay utilizing a luminometer [12].

The amount of ATP is quantified in Relative Light Units (RLU) through the enzymatic conversion of ATP into light. As a result, a high RLU readout indicates cellular contamination, thereby fostering the growth and dissemination of microorganisms [13]. RLU are commonly used to quickly assess surface cleanliness in healthcare and other settings [14]. The ATP bioluminescence method has gained popularity due to its simplicity and rapidity, addressing the delays associated with the colony counting method [12]. Nonetheless, the relationship between the measured ATP levels and microbial contamination within healthcare and other settings is limited, and diverse studies present varying correlations [12, 13, 15–17].

In this study, we investigated, for the first time, the potential correlation between ATP content and microbial load on spectacle surfaces. In addition, six different types of widely used spectacle cleaning approaches were assessed for their cleaning efficacy. Overall, our study aimed at establishing ATP measurements as a rapid method for evaluating the microbiological cleanliness of spectacles, which can easily be used by non-experts outside a microbiological laboratory, such as by opticians in an optician’s store or during a trade fair for ophthalmic optics.

## Methods

### Evaluation of different cleaning methods for spectacles by ATP bioluminescence method

A total of 30 worn spectacles were voluntarily provided by staff and students of Furtwangen University, campus Villingen-Schwenningen. Cleaning tests were performed with half spectacles, including the total area (front and back, respectively) of one lens, nose pad and ear clip, estimated to have a total surface area of 30 cm^2^. Per cleaning procedure, ten spectacle halves stemming from different spectacles were used, i.e. the sample size was 10 per investigated procedure. ATP measurements were conducted with a Kikkoman Lumitester PD-30 (Kikkoman Biochemifa, Tokyo, Japan), utilizing LuciPac pen swabs (Kikkoman Biochemifa), with results reported in RLU. Based on the study by Bakke [7], 100 fmol of ATP correspond to an RLU value of 174 using this Kikkoman technology. All measurements were performed by the same person following the manufacturer's guidelines. Using sterile swabs yielded RLU values between 0 and 5. Pre-cleaning ATP measurements were carried out with the uncleaned, worn spectacles. Subsequently, the identical area underwent standardized cleaning. After that, post-cleaning ATP measurements were taken. During each cleaning procedure, the respective spectacle surfaces were wiped five times upward and five times downward with the respective cleaning device. For each cleaning, a new device was used.

The study evaluated six different cleaning materials for their effectiveness. The first method involved a dry cotton towel (TW). The second method utilized a soap water (SW) solution, created by mixing 2 drops of regular dishwashing liquid with 2 ml of tap water. This solution was generously applied to all surfaces of the spectacle, followed by standardized wiping with a dry cotton tissue. The third material tested was a dry, finely textured microfiber cloth (MC), while the fourth consisted of dry cotton tissues (DCT), which were cellulose-based and alcohol-free. The fifth method utilized antibacterial wipes (ABW) infused with ethanol and isopropanol, and the sixth used alcohol-free wipes (AFW) containing an amine-based cleaning solution. Except for the soap water solution, all cleaning materials were supplied by Carl Zeiss Vision International GmbH (Aalen, Germany).

### Corelation between germ count and ATP content

10 spectacles were provided voluntarily by employees and students of Furtwangen University, campus Villingen-Schwenningen. Each spectacle was divided into two halves, with one half allocated for ATP measurement, while the remaining half was designated for colony counting, assuming no difference in microbial load between the right and left halves. Indeed, small scale analyses indicated no significant differences of the ATP content on the left (150 ± 37.01 RLU/cm^2^) and right sides (121.66 ± 17.56 RLU/cm^2^) of 5 worn spectacles. The same was true for the aerobic germ counts (left: 123 ± 34 CFU/cm^2^, right: 124 ± 54 CFU/cm^2^) and anaerobic germ counts (left: 77 ± 74 CFU/cm^2^, right: 160 ± 51 CFU/cm^2^) obtained with 3 worn spectacles (median ± mean deviation from median). All samplings took place in the university laboratory. ATP measurements were performed as described above. Germ counts were performed as previously reported [4]. In brief, each spectacle half was sampled twice with sterile cotton swabs, first with a wetted swab and then with a dry swab. A sterilized medium containing 1.5 g of casein peptone (Carl Roth, Karlsruhe, Germany) and 12.75 g of sodium chloride (Carl Roth) per 1500 mL of water were utilized for the wet swab. This medium was also used for the subsequent dilution series. After the sampling process, the wet and dry swab heads were combined and vortexed for 10 s in 2 mL of wetting medium to suspend all microbes.

Germ quantities were determined from the suspension through serial dilution up to 10^–5^, and 50 µL of each dilution was plated in duplicates on Tryptic Soy Agar plates (TSA; Carl Roth), serving as a non-selective medium for bacterial cultivation. The plates were then incubated under aerobic conditions for 3 days at 37 °C. Thioglycolate Agar (Merck, Darmstadt, Germany) was used for bacterial cultivation under anaerobic conditions for a duration of 10 days at 37 °C. The anaerobic cultivation was conducted in an anaerobic jar utilizing an anaerocult with indicators (Merck). Colonies within the range of 30 to 300 per plate were counted. Germ numbers were expressed as colony forming units (CFU) cm ^−2^. Sterility tests showed no growth when an inoculum was absent.

### Statistical analysis

To assess the effectiveness of different cleaning methods, median RLU values and mean deviations from the median (MDM) were calculated. The statistical analysis was done using R (version 4.3.0) [18] and R Studio (version 2023.09.1 + 494) [19]. Normal distribution was assessed using the Shapiro–Wilk test. As not all data followed a normal distribution, non-parametric tests were applied. The Mann–Whitney-U test was used to compare pre- and post-cleaning RLU values. The Kruskal–Wallis-H test was conducted to compare the relative reduction of RFU values by the different cleaning techniques. Subsequently, post-hoc pairwise comparisons were conducted. The correlation between RLU and CFU values was calculated using scatterplots and the Pearson correlation coefficient.. Results with adjusted p-values < 0.05 were considered statistically significant.

## Results

### Effect of different cleaning methods

Table 1 shows the efficacy of the tested cleaning approaches in reducing contamination on spectacles, as measured by ATP levels. The assessment is based on pre- and post-cleaning ATP levels, with percentage-based reduction and corresponding p values providing insights into the efficacy of each method.

**Table 1:** Effect of different cleaning methods on ATP content as measure for contamination of spectacles

Cleaning Approach	Pre-cleaning ATP median ± MDM (RLU/cm^2^)	Post-cleaning ATP median ± MDM (RLU/cm^2^)	Median relative reduction ± MDM (%)	Adjusted p value
Towel (TW)	74.98 ± 26.56	11.10 ± 8.25	84.08 ± 7.95	0.000043
Soap water (SW)	45.03 ± 70.14	9.23 ± 9.10	74.78 ± 20.46	0.002089
Microfiber cloth (MC)	256.63 ± 232.17	30.87 ± 37.83	84.44 ± 8.82	0.011300
Dry cotton tissue (DCT)	385.57 ± 344.80	26.38 ± 49.27	84.65 ± 10.72	0.002879
Antibacterial wipe (ABW)	66.83 ± 77.94	9.87 ± 7.24	88.84 ± 3.96	0.000010
Alcohol free wipe (AFW)	70.77 ± 42.30	5.68 ± 4.67	93.00 ± 8.37	0.000370

To investigate the effect of cleaning, 10 spectacle halves (ca. 30 cm^2^) were used for each cleaning experiment (MDM, mean deviation from median; RLU, relative light unit)

All cleaning methods significantly reduced ATP levels between 75 and 93%, indicating effective cleaning performance. Statistical analysis showed an overall significant difference between the different cleaning methods, when based on relative reduction of ATP level (p = 0.01335). However, pairwise comparisons only revealed that AFW was significantly more effective than both DCT and MC, with p values of 0.03 and 0.04, respectively. No other pairwise comparisons were found to be significant. Nevertheless, formulated as a trend, the observed methods might be grouped into methods showing > 85% reduction (AFW, ABW), 80–85% reduction (DCT, TW, MC) and less than 80% reduction (SW).

### Correlation between germ count and ATP content

**Fig. 1:**
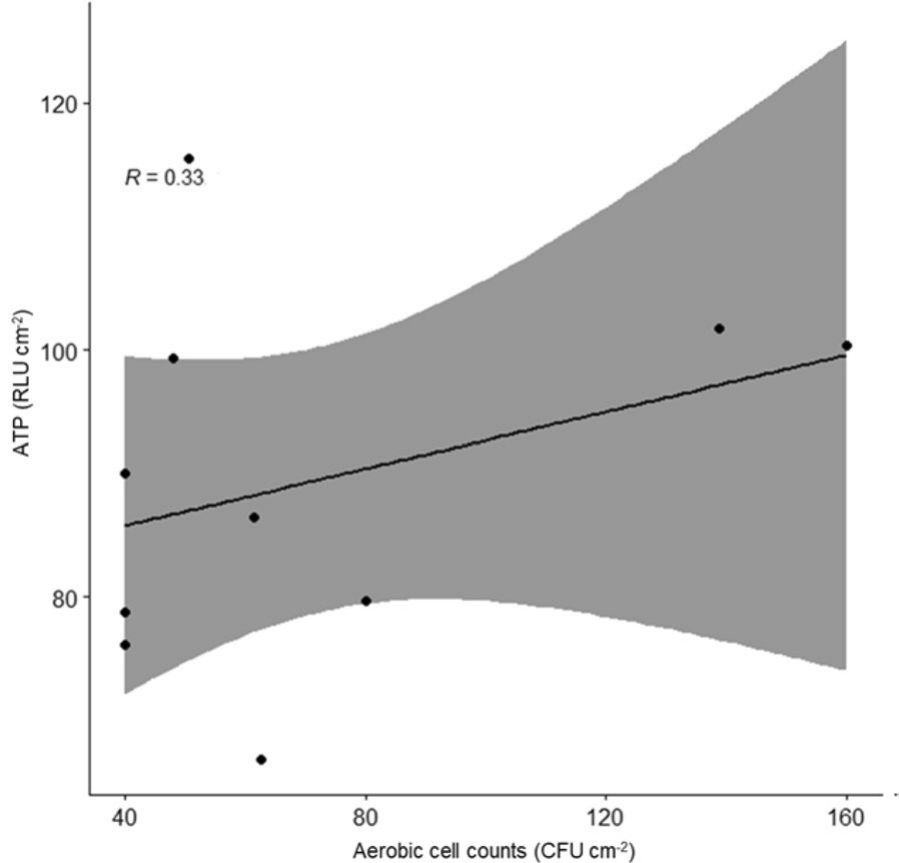
Scatterplot illustrating the correlation between aerobic germ counts and ATP. The correlation was not significant (p = 0.36). The line of best fit for the data (95% confidence interval) is within the shaded area. R = correlation coefficient

**Fig. 2:**
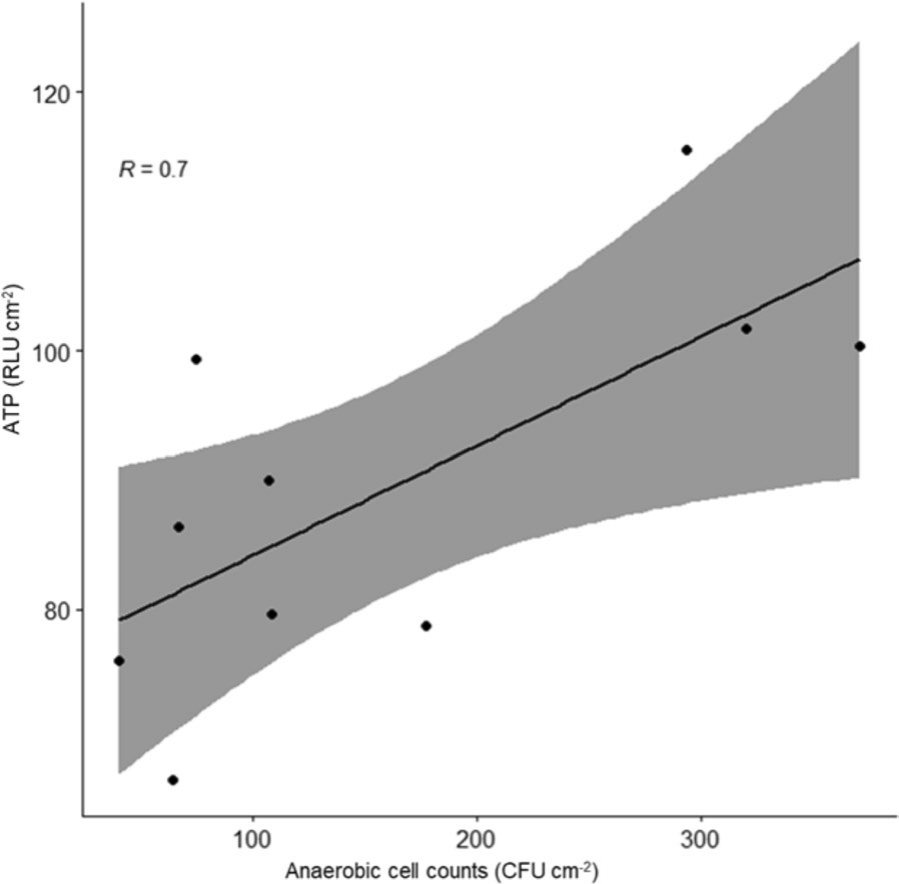
Scatterplot illustrating the correlation between anaerobic germ counts and ATP levels. The correlation was significant (p = 0.025). The line of best fit for the data (95% confidence interval) is within the shaded area. R = correlation coefficient

In Figs. 1 and 2, ATP levels measured in RLU cm^−2^ and germ counts in CFU cm^−2^ are plotted against each other.

Figure 1 shows that the aerobic CFU values ranged from a minimum of 40 to a maximum of 160 CFU cm^−2^, indicating varying levels of aerobic bacterial contamination across different spectacles. Values of the ATP measurements ranged from 66.83 to 115.57 RLU cm^−2^. The median values were 56 CFU cm^−2^ for aerobic germ count and 88.22 RLU cm^−2^ for ATP. The correlation between aerobic CFU and ATP levels was further analysed in the statistical analysis, with a p-value of 0.34 suggesting no corelation.

In Fig. 2 anaerobic germ counts and ATP measurements are presented. Anaerobic CFU values ranged from a minimum of 40 to a maximum of 371 CFU cm^−2^, showcasing a broad spectrum of anaerobic bacterial contamination. The median was 107 CFU cm^−2^ for anaerobic germ count. ATP data were as reported above. The correlation between germ counts and ATP levels was found to be significant with a p-value of 0.025.

## Discussion

The aim of the study presented here was to investigate the suitability of ATP measurements to demonstrate the efficacy of popular cleaning measures for worn spectacles and to investigate possible correlations between germ counts and ATP content on spectacle surfaces.

Despite its frequent use, an actual correlation between ATP content and microbial contamination could not be proven for all settings [20, 21]. Nevertheless, some researchers previously reported a correlation between ATP and CFU on surfaces [22–25]. Our data also show a significant correlation between ATP content and anaerobic germ counts. Notably, oxygen-tolerant anaerobes (e.g., cutibacteria) were identified as the most abundant taxa in our previous studies on spectacles [26] and similar surfaces [6], which might explain that only a significant correlation with anaerobic but not with aerobic germ counts was found. In addition, the correlation analysis also might have been influenced by non-microbiological ATP-sources, such as human skin cells or skin-borne ATP. Finally, the aerobic colony count method used here also has some limitations, such as incubation time, temperature and the choice of growth medium [27]. The use of TSA agar plates cultured at 37 °C for 72 h might have discriminated slow-growing microbes, those with more specific nutrient requirements or environmental bacteria with a temperature optimum below 37 °C [13]. This will be considered in follow-up studies. Nevertheless, the observed link between ATP content and germ counts indicates that ATP measurements are suitable to rapidly evaluate the microbiological hygiene status of spectacles surfaces and the efficacy of cleaning measures.

All investigated cleaning techniques lead to a significant reduction of the ATP levels on spectacle surface, indicating that all methods are suitable to improve the hygiene status of spectacles [28]. However, due to the large dispersion of the data and the relatively small number of replicates, there were hardly any significant differences between the six investigated cleaning techniques. In general, the reduction of ATP might be caused by the mechanical removal of cells and ATP molecules from the smooth, and hence easy to clean surfaces, aided by detergents, solvents and antimicrobial actives in the respective products.

The tested non-alcoholic wipes led to significantly better ATP removal than cotton tissue and microfabric cloth. Similar results were obtained in our previous study [4] in which we investigated the removal and inactivation of test bacteria from glass lenses with dry and wet lens wipes. Notably, choosing non-alcoholic, tenside-based products for cleaning spectacles can be regarded beneficial from a technical point of view, as it protects delicate spectacle parts, such as plastic frame material, from potential damage by alcohol [4]. Though not statistically significant, the relatively bad performance of soap water followed by drying with a cotton tissue, was not expected and needs to be validated in further studies, e.g. by improving the cleaning procedure itself. Nevertheless, considering the detergent and antimicrobial properties of soap and it its easy accessibility, also cleaning with soap water can be regraded an effective measure to improve the hygienic status of spectacle surfaces [29].

## Conclusion

The microbial load on spectacle surfaces is correlated with its ATP content. ATP measurement can be used as an easy-to-use and rapid method to demonstrate the efficacy of spectacle cleaning measures. It can also be used by non-microbiologists and also under non-laboratory condition, such as in optician’s shops or during a trade fair for ophthalmic optics, to demonstrate the efficacy of (new) cleaning measures. The evaluation of six widely used spectacle cleaning approaches revealed a significant reduction in ATP levels between 75 and 93%, indicating all methods suitable to improve the hygienic status of spectacles. Future research can include additional cleaning methods (such as ultrasonication or UV-C irradiation) and should aim at establishing probably situation-dependent borderlines, which germ counts and ATP contents on spectacle surface are tolerable from a hygienic point of view.

### Limitations

Our study has limitations with respect to the small sample size of investigated worn spectacles, which were all stemming from a single environment, i.e., a university environment. In addition, only two cultivation media were used for aerobic and anaerobic cultivation, respectively, which might have hampered the detection and quantification of a broader variety of bacteria. Similarly, only a single ATP detection technology was used. All cleaning tests were standardized and performed by a single person. However, it cannot be excluded that in the hands of other persons, the tested methods might yield different cleaning results. Finally, our data do not provide any thresholds, from which ATP content or microbial load on, spectacle surfaces might be regarded as safe from a hygienic point of view.

## Data Availability

All data are available from the corresponding author upon reasonable request.
